# Vocal registers expand signal diversity in vertebrate vocal communication

**DOI:** 10.1098/rstb.2024.0006

**Published:** 2025-04-03

**Authors:** Christian T. Herbst, Coen P. H. Elemans

**Affiliations:** ^1^ Bioacoustics Laboratory, Department of Cognitive Biology, University of Vienna, Vienna, Austria; ^2^ Department of Communication Sciences and Disorders, College of Liberal Arts and Sciences, University of Iowa, Iowa, IA USA; ^3^ Sound Communication and Behavior Group, Department of Biology, University of Southern Denmark, Odense, Denmark

**Keywords:** vocal communication, MEAD, saddle-node bifurcation, motor control, vibratory state

## Abstract

Among air-breathing tetrapods, the most common sound production mechanism is flow-induced self-sustained tissue oscillation, aka voiced sound production, driven by inherently nonlinear physical processes. Some signature features like deterministic chaos have received particular attention in bioacoustics as nonlinear phenomena (NLP). However, one type of NLP that extends frequency ranges and enriches timbres has received much less focus in comparative bioacoustics: vocal registers. Controlled by muscle activity, vocal registers constitute distinct periodic vibratory states of vocal tissues. Transitions between vocal registers often lead to abrupt fundamental frequency jumps, which are, e.g., deliberately used in human alpine yodelling, for example. Theoretical work suggests that register transitions are caused by saddle-node-in-limit-cycle bifurcations. Here, we review the biophysical underpinnings of vocal registers and what signatures they leave in vocal fold kinematics and acoustics in the best studied species: humans. Apart from human speech and song, registers have been described only in a few animal taxa, but the occurrence of signature features suggests that vocal registers could be much more common across vertebrates than currently appreciated. We suggest that registers are a fundamental trait of voice production and that they are favoured in selection because they vastly extend and diversify the acoustic signalling space.

This article is part of the theme issue ‘Nonlinear phenomena in vertebrate vocalizations: mechanisms and communicative functions.’

## 1. Background

Voiced sound production is generated by flow-induced self-sustained oscillations of tissues according to the myo‑elastic aero‑dynamic theory (MEAD) [1]. This MEAD mechanism has been adapted multiple times independently to function in distinct anatomical structures including mammalian vocal folds (VFs) and ventricular folds (see e.g. [2]), avian syringeal labia [3] and nasal phonic lips in toothed whales [4]. Although perhaps conceptually simple, the mechanism involves complex physical processes, including fluid flow, structural mechanics of soft tissues, acoustics and their interactions. These processes are inherently nonlinear, hence the successful application of nonlinear dynamical theory to voice production (see this issue).

In animal voice production, several types of abrupt changes are commonly observed that can be explained with concepts of nonlinear dynamics. The most common and crucial state change in sound production is the transition from non-oscillation (silence) to self-sustained oscillation (sound) of VF, or analogue structures. For example, when the bronchial pressure or a muscle’s tension slowly changes, it can reach a critical point where—in dynamical terms—the stability of the system switches from a fixed point to a periodic solution and becomes a small amplitude limit cycle (LC) [5,6]. This bifurcation is called the Hopf bifurcation [7,8] as explained in detail elsewhere in this issue.

In the human voice, another bifurcation phenomenon is also very prevalent in both speech [9] and singing [10]: the transition between so-called vocal registers. Registers are distinct vibratory regimes of the VFs that lead to distinct frequency range and spectral content of each register. For the human voice, at least three well-known vocal registers, or ‘laryngeal mechanisms’ (M0–M3) are recognized: vocal fry (M0), chest (M1), falsetto voice (M2) and ‘whistle register’ (M3) [11,12]. Registers greatly enhance the overall frequency range and spectral diversity of human voice production and are exploited heavily in artistic expression.

Vocal registers in humans were acknowledged as early as the thirteenth century [13], but received only relatively little attention in bioacoustics. Recently, the occurrence of multiple vocal registers has been shown or suggested in several mammals including several non-human primates, dogs, cats and toothed whales, and birds (see table 1). Thus, the occurrence of vocal registers seems more common than previously thought. In the species investigated to date, multiple registers seem to particularly expand signal diversity. In some extreme cases such as toothed whales, vocal registers not only form the basis for their large vocal repertoires, but one of the registers—vocal fry—allowed novel ecological functionality of vocal communication and drove the evolution of underwater echolocation [4].

**Table 1.** Overview of previous works suggesting the existence of vocal registers in nonhuman vocalizations. We included the original register vocabulary as used by the authors and additionally applied the modern classification into M0–M3 based on our interpretation of the data, insofar as possible. M0 (‘vocal fry’ or ‘pulse’), M1 (‘chest’ or ‘modal’), M2 (‘falsetto’, ‘head’ or ‘loft’) and M3 (‘whistle’ or ‘flageolet’). Note that in some cases, there is only a vague contextual link between the described ‘register’ and the modern register classification of M0 through M3. LEMG: laryngeal electromyography; EGG: electroglottography; HSV: laryngeal high-speed video.

source	publication year	species	expt. setup	registers (lit. source)	lar. mech.	evidence and interpretation
Solomon *et al.* [14]	1994	dogs (*n* = 6)	*in situ*	‘growl’ = vocal fry; ‘howl’ = modal, chest or head	M1, M2	LEMG, acoustic, tracheal pressure (perceptual assessment)
Brown & Cannito [15]	1995	Sykes’s monkeys (*n* = 2)	*in vivo*	‘squeal’ = ‘modal’ or ‘falsetto’	M1, M2	EGG, acoustic (EGG interpretation)
Berry *et al.* [16]	1996	dogs (*n* = 5)	*in vitro*	chest, falsetto and flageolet/whistle register	M1, M2, M3	acoustic, subglottal pressure (perceptual evaluations and qualitative differences in spectra)
Tembrock [17]	1996	‘mammals, various taxa’ (*n* = unknown)	*in vivo*	falsetto	M2	unknown (‘discontinued jump’ of fundamental frequency)
Riede *et al.* [18]	1997	Japanese macaques (*n* = 10)	*in vivo*	N/A	N/A	acoustic (‘sudden changes in the fundamental frequency’)
Wilden *et al.* [19]	1998	common dormouse, African wild dog (*n* = 1)	*in vivo*	modal and falsetto register	M1, M2	acoustic (sudden transition between limit cycles with different amplitude and fund. freq.)
Riede & Zuberbühler [20]	2003	Diana monkeys (*n* = unknown)	*in vivo*	pulse register	M0	acoustic (similarity of fundamental frequency with human ‘pulse register’)
Jensen *et al.* [21]	2007	hooded crows (*n* = 3)	*in vivo*/*in vitro*	pulse register	M0	acoustic, thoracic pressure, HSV (acoustic and the videographic evidence similar to vocal fry in humans)
Riley *et al.* [22]	2016	lambs (*n* = 8)	*in vivo*	N/A	N/A	acoustic (‘episodes of amplitude variability’)
Rasmussen *et al.* [23]	2018	pigeons (*n* = 8)	*in vitro*	N/A	N/A	acoustic, EGG, HSV, subglottal pressure (distinct EGG waveforms for high- and low-frequency vocalizations)
Herbst *et al.* [24]	2018	Japanese macaques (*n* = 2)	*in vitro*	‘coo’ = falsetto	M2	acoustic, EGG, HSV, subglottal pressure (EGG evidence)
Herbst *et al.* [25]	2023	cats (*n* = 8)	*in vitro*	vocal fry	M0	acoustic, EGG, HSV, subglottal pressure (oscillatory closed quotient, fundamental frequency)
Madsen *et al.* [4]	2024	Atlantic bottlenose dolphin (n = 3), harbor porpoise (n = 7), sperm whale (n = 2), false killer whale (n = 1)	*in vivo*/*in vitro*	M0, M1, M2	M0, M1, M2	acoustic, HSV, sub- and supraglottal pressure (vocal fold acceleration, vibratory open quotients)
Schlegel *et al.* [26]	2024	dogs (*n* = 1)	*in situ*	chest, falsetto	M1, M2	LEMG, acoustic, HSV, subglottal pressure (fast and sudden jumps or drops of fundamental frequency)
Herbst *et al.* [27]	2025	New World monkeys (*n* = 6)	*in vivo*/*in vitro*	register-like transitions	N/A	acoustic, EGG, HSV, subglottal pressure (EGG analysis: abrupt changes of fundamental frequency)

Here, we explore if registers could be a common trait in the vertebrate voice and be used to increase signal diversity. We review the current state-of-the-art insights in the production of vocal registers in the best studied species, humans, followed by the types of evidence that have been used to support the occurrence of register transitions in other animals. The bioacoustics literature shows that frequency jumps—a signature of register transitions—can be found in the voices of animals in all major clades of vertebrates. We suggest that vocal registers are an intrinsic feature of voiced sound production that vastly increase signal diversity in the voice production in all vocal vertebrates.

## 2. Vocal registers in the human voice

Because early research in human voice did not have experimental access to the physiology and physics of voice production, it is thus not surprising that, historically, the first attempts to classify vocal registers in humans were either influenced by perceived proprioception—as in the ‘head voice’ (Ital.: *voce di testa*) versus the ‘chest voice’ (Ital.: *voce di petto*) [28]—or focused on the radiated sound as ‘perceptually distinct regions of vocal quality that can be maintained over some ranges of pitch and loudness’ [29].

The number of vocal registers in humans remains debated [12], but a trend emerges for contemporary sources to consider four registers: M0 (also termed ‘vocal fry’ or ‘pulse’ register), M1 (‘chest’ or ‘modal’), M2 (‘falsetto’, ‘head’ or ‘loft’) and M3 (‘whistle’, ‘flute’ or ‘flageolet’) [30]. While M1 is used as the predominant vocal register for speech production, singing is mostly produced with the M1 and M2 registers [31]. M0 [32] is used in both speech [33,34] and as an effect in singing [35], and M3 seems to be exclusively utilized in high-pitched singing [36,37] and perhaps during spontaneous non-verbal communication.

When considering the radiated sound pressure, two features of vocal registers are important: Most crucially, within an individual, fundamental frequency ( $f_{o}$ ) ranges are ordered from low to high, with M0 covering the lowest and M3 covering the highest $f_{o}$ part of the total range. Overlaps of the individual registers’ $f_{o}$ ranges are possible to some degree [30,31,38]. Transitions from one register to another are often accompanied by abrupt alterations of $f_{o},$ termed ‘frequency jumps’ or ‘pitch jumps’ [39–41]. A secondary acoustical trait of vocal registers is their distinct spectral composition [42–44], which may, however, vary considerably across individuals.

For the purpose of this review, we here use the term ‘vocal registers’ for different vibratory states of the sound-generating tissues, instead of ‘laryngeal mechanisms’ as often used in humans [11]. We did this because in vertebrates, sound production is not limited to voiced sound generation, but also includes, for example, aeroacoustic whistles in rodents [45], gas bladder pulsation by muscle contractions in fish [46] and excitation of laryngeal resonance modes in frogs [47], to name a few. These represent different sound production mechanisms at large. For this reason, the term ‘laryngeal mechanism’ used in humans does not cover the much larger diversity of vocal registers present in animal bioacoustics. Furthermore, given vastly different laryngeal anatomy and morphology of the sound-generating tissues across the vertebrates, it is likely that more than the four categorical vibratory states (M0–M3) can be found in species other than humans.

In humans, the production of the different registers has been shown to be influenced by two different sub-systems of the vocal apparatus: (a) the laryngeal voice source and (b) the supra- and subglottal vocal tracts.

### (a) Vibratory states of human vocal folds

**Figure 1.**
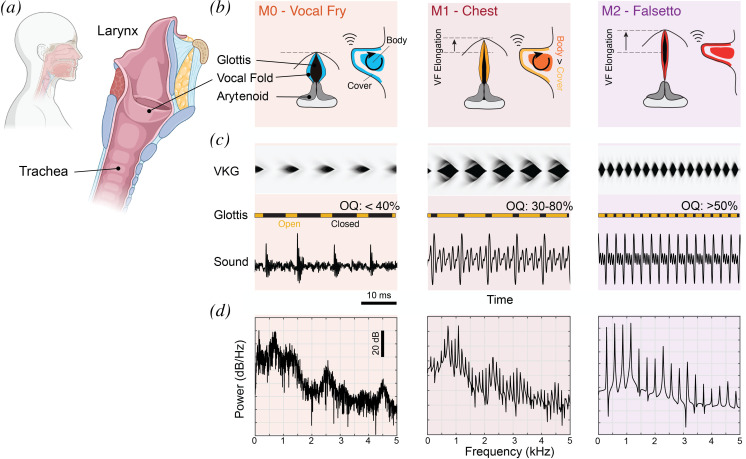
Hallmarks of voice registers in human speech and song. (*a*) Overview of the human larynx in sagittal view. Drawn with Biorender. (*b*) Anatomical top and side view of the vocal folds of the three common voice registers in humans: M0, M1 and M2. (*c*) Prototypical oscillation of the vocal folds showing simulated videokymographic imaging (VGK; top), open and closed phase of the glottis (middle) and associated sound pressure (bottom) as a function of time. See the text for explanation. VKG images from [49]. Note that M0 VKG data represent ‘pressed voice’ in [49], but this can be expected to look very similar to M0. (*d*) Sound spectra of signals in the panel using the periodogram method.

The complex vibratory behaviour of the VFs is in essence a tissue resonance phenomenon driven by air flow [48]. Gradual changes in VF posture by laryngeal muscles change the synchronization of VF resonance modes or eigenmodes that lead to self-sustained VF oscillations and bifurcations between the different vocal registers. Distinct levels of muscle activity change (i) the overall posturing of the VFs and (ii) the differential stiffness of and tension within outer (cover) and inner (body) VF tissue layers (see figure 1). The following four vocal registers have been documented in humans:

— **M0** (‘vocal fry’ or ‘pulse register’). The VFs are short and slack. This results in the lowest possible oscillatory frequencies with a very short open phase of the vibratory cycle (open quotient (OQ): 0–0.4). Glottal air flow rates are very low [50] and VF acceleration and tracheal sound are pulsatile [51,52]. Vocal fold length is typically lower than in M1, and—in contrast to M1—no correlation is found between VF length and $f_{o}$ [53], suggesting a fundamentally different mechanism for $f_{o}$ control as compared to the other registers, which requires further investigation. During M0 vibration, there is low activity in the cricothyroid (CT) and interarytenoid muscles and increased thyroarytenoid (TA) muscle activity [54], as compared with M1.
— **M1** (‘chest’ or ‘modal’ register), the main register used in human speech, is—in contrast to M2—typically induced by a certain degree of TA muscle activation while relatively relaxing the CT muscle [55], leading to a thickening, shortening and medial bulging of the VFs [38,56]. This increases the vertical thickness of the VF [57] and is crucial for the establishment of M1. There is a higher degree of tension in the VF body compared with the VF cover [58], allowing a certain degree of vibratory independence of these two VF portions. This typically results in a so-called mucosal wave in the VF cover [59], i.e., a wave travelling in the inferior-superior and then lateral direction along the VF edges [60]. A mucosal wave introduces a vertical phase delay of about one-sixth to one-quarter of the vibratory cycle [61] into the VF vibration, thus setting up a rotational vibratory component (or rotational vibratory mode) [62,63]. Generally, M1 is characterized by relatively stronger rotational vibratory components as compared with M2 [64]—see [2] for further discussion.
— **M2** (‘falsetto’, ‘head’ or ‘loft’) is often portrayed as the antagonist to M1 and is typically produced with a lesser degree of TA activation and a relatively stronger CT muscle contraction. The VFs are further lengthened with both body and cover stiff leading to higher frequencies and OQ between 0.5 and 0.95 [65]. There is a greatly reduced mucosal wave.
— **M3** (‘whistle’, ‘flute’ or ‘flageolet’) is perhaps the least understood register of the human voice because multiple phenomena may have been labelled with this term. At least for the upper $f_{o}$ of classical/operatic singing, an aeroacoustic (i.e. ‘whistle’) production principle has been ruled out recently, showing that this register is produced in a manner similar to M2 [36]. Potentially, the VF tension is greatly increased, but this remains unknown. Apart from laryngeal phenomena, the special ‘flute-like’ perceptual timbre of this vocal register may be caused by a distinct vocal tract resonance tuning strategy [66,67] rather than by a special laryngeal vibratory state. There are, however, also suggestions of a true aeroacoustic voice production phenomenon without VF vibration in ultra-high $f_{o}$ in human vocalizations [68–70], but further research is needed to confirm this notion.

The different oscillatory profiles of the laryngeal vibratory states have a tendency for increasingly smaller durations of intra-cycle VF contact when going from M0 to M3, with OQ values somewhat correlating with $f_{o}$ . Given the causal connection of OQ and the strength of higher harmonics within the acoustic voice source [71,72], there is a tendency for the lower registers to result in a radiated sound with stronger higher harmonics—see figure 1*c*,*d*. In contrast, higher registers have a tendency to have weaker upper harmonics and a relatively stronger fundamental (but note that there is a high degree of individual variation, depending not only on VF morphology but potentially also on key phonatory parameters such as subglottal pressure [73,74] or the degree of VF adduction [38,75]). Within limits, this thus allows in distinguishing between the different registers in humans, based on the acoustic output.

### (b) Vocal tract influence on registers

A recent study has shown that transitions between vocal registers can in principle be induced through laryngeal (i.e. VF) dynamics alone, without any influence of the subglottal or supraglottal vocal tract [41]. However, the vocal tracts may play an additional role in establishing vocal registers and their transitions in two cases.

(1) When a voice source harmonic is close to a vocal tract resonance in the frequency domain, the amplitude of that particular harmonic can be greatly increased via resonance effects of the supraglottal vocal tract, according to the linear source–filter theory [76]. This is called formant tuning [77] and occurs in several types of vocalizations, including human singing [78], vocalizations of non-human mammals [79] and birds [80–82]. Such strengthening of radiated harmonics results in distinct changes in the radiated acoustic spectrum [10,83], which may be perceived as register changes even without change of laryngeal vibratory state (see fig. 1 in [12] for an example).
(2) In addition, nonlinear interactions between vocal tract and source—already predicted about 100 years ago [84]—may lead to abrupt alterations of the VF periodicity and dynamics of laryngeal tissue oscillation, resulting in register changes of $f_{o}$ . These frequency jumps are theorized to occur when a strong voice source harmonic is passing through a vocal tract resonance [85]. Empirical data suggest that this may happen when changing either phonatory $f_{o}$ [86,87] and/or the produced vowel [88], likely depending on the coupling strength between vocal tract and voice source [1]. Notably, such effects can potentially be induced by both the supraglottal and the subglottal vocal tract [89,90]. Inasmuch such source–tract interactions can facilitate actual transitions between vibratory states [8] and/or whether they introduce additional bifurcation points independent of laryngeal vibratory states in the $f_{o}$ range requires further investigation.

## 3. Vocal registers in the context of nonlinear dynamics

Because vocal registers represent distinct vibratory states, they - and transitions between them - are best analysed in view of nonlinear dynamics. Therefore, we first present a brief, comprehensive review of nonlinear dynamics, focusing on human voice register theory [8,16,91]. Voice is a system of coupled oscillators [92]. It typically involves one or more sets of paired, coupled oscillators (VFs, ventricular folds, aryepiglottic folds, syringeal VFs in birds, etc.) and an upstream (subglottal) and downstream (supraglottal) vocal tract. The spatio-temporal dynamics of the viscoelastic materials, the air flow through the glottis and the vocal tract resonators are governed by pronounced nonlinearities. Depending on biophysical boundary conditions, which are set by the physiological variables of vocalization (including tracheal/subglottal air pressure and activation of intrinsic laryngeal muscles), each of the involved oscillators has its own distinct set of eigenfrequencies or resonances [93]. Partly depending on the coupling strength between the involved vibratory components, these eigenfrequencies may be entrained to produce stable oscillation (periodic or subharmonic) within the system at large—see [2] for a detailed discussion.

A stable solution—either stationary or non-stationary—within the phase space of a nonlinear dynamic system is called an attractor. An attractor is the final state of a dissipative dynamical system that is approached over time for given boundary conditions and initial values when the supplied and the dissipated energy are balanced [94]. The attractor emerging for periodic oscillation is called a limit cycle (LC). Laryngeal vibratory states in vertebrate vocalization are typically produced by (nearly) periodic oscillation, but chaotic oscillation is not considered a vocal register. For an individual during a given vocalization, each vibratory state (vocal register M0–M3) is represented by a distinct and characteristic LC.

**Figure 2.**
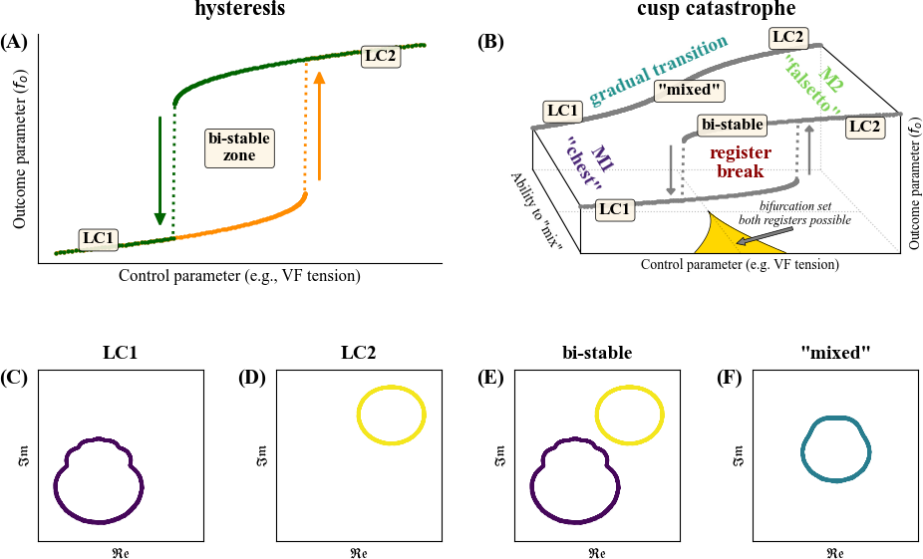
Illustration of nonlinear dynamics theory for voice register transitions. (A) Hysteresis of $f_{o}$ control (inspired by [39], (). (B) cusp catastrophe representation of a register transition, inspired by [94]. (*C–*F) Phase space diagrams for different system states within the cusp catastrophe model. Limit cycle **LC1** (panel C) represents vocal register M1 and **LC2** (panel D) represents vocal register M2. **bi-stable** (panel E): co-occurrence of LC1 and LC2 in the hysteresis scenario in the intermediate $f_{o}$ range. ‘**mixed**’ (panel F): scenario that supports a gradual transition between LC1 and LC2. Please refer to electronic supplementary material, movies S1 and S2 for an animated version of this figure.

Nonlinear dynamics register theory suggests that a transition between vibratory states can be either achieved (i) with an abrupt alternation between different LCs; or (ii) in a gradual way, thus ‘mixing’ or ‘blending’ the registers (figure 2), which can be explained as follows.

Voice production has the potential for multiple stable attractors which coexist in the system’s state space. There is typically a certain overlap between the individual registers’ $f_{o}$ range. When the $f_{o}$ ranges of two registers overlap, both registers could theoretically be produced. In such a case, the actually emerging register is determined by the system’s previous state, resulting in a so-called hysteresis phenomenon. This is schematically illustrated in figure 2A. In the range of $T_{A}..T_{B}$ only limit cycle LC1 is possible (representing M1 in the illustrated case [39]). When longitudinal VF tension T is increased beyond $T_{B}$ , the system enters a bistable zone where both LC1 (M1) and LC2 (M2) would be possible. However, because the previous system state was LC1, the system stays in LC1. Only when T is increased beyond $T_{C}$ will the system abruptly jump to limit cycle LC2, which represents M2 in the given example, because LC1 is no longer supported by the system when $T>T_{C}$ . Such a transition typically results in an abrupt and clearly measurable frequency jump. When operating the system with VF tension in the range of $T_{C}..T_{D}$ , the system will always oscillate in LC2. If from this starting point the tension is then reduced below $T_{C}$ , the system again enters the bistable state, but this time staying in LC2. A return to LC1 is in that condition only possible if T is reduced below $T_{B}$ . Consequently, at what point in the state space, the bifurcation occurs depends on the history of the behaviour and this phenomenon is called hysteresis.

The scenario illustrated in figure 2A is only one theoretical possibility for transitioning between registers. In humans, other paths are possible if the singer manages to adequately optimize the vocal system’s parameters, e.g. adapting the contraction of the intrinsic laryngeal muscles (thereby potentially changing the influence of certain mechanical oscillatory modes within the VFs) or optimizing the resonance structure of the supralaryngeal vocal tract (thereby potentially creating favourable feedback and interaction conditions between filter and source). The multitude of different pathways for transitions from one to another register is depicted in figure 2B, which represents a cusp catastrophe in terms of nonlinear dynamics [94,95]. Essentially, figure 2B is a schematic illustration of figure 2A, but augmented by a third dimension (labelled ‘ability to mix’). A case of smoothly blended registers is depicted in the back of that graphic. The different prototypical scenarios for phase space attractors and LCs are shown in figure C–F—see also electronic supplementary material, movies S1 and S2 for an animated illustration. Panels C and D represent the stable conditions where either limit cycle LC1 (M1) or LC2 (M2) are exclusively supported by the system. Panel E illustrates the bistable zone where both LC1 and LC2 are possible. This bistable zone is depicted with a yellow triangle at the bottom of figure 2B. Finally, panel F illustrates the state of a smooth transition which allows for a gradual merging between LC1 and LC2, resulting in a ‘mixed voice’ or ‘voix mixte’ [92]. In nonlinear terms, the bistable zone of a register transition is supported by saddle-node bifurcations [94–96], also referred to as SNILC (saddle‑node bifurcation in limit cycle) [91] or SNLC (saddle-node limit cycle bifurcation) [97].

The saddle-node (SN(I)LC) bifurcation mentioned above has been used successfully to describe vocal register transitions in low-dimensional mass models of the VFs [98–103] in both humans [101] and birds [100,102]. Please note that the human and birdsong fields use a slightly different terminology for SNILC bifurcations (see [104]). Just as in human voice models, in both single mass [102] and two-mass [100] models of avian voice production, SNILC bifurcations changed both collision force and harmonic content.

## 4. Vocal registers extend the frequency range

The different vocal registers occur in distinct, perhaps overlapping, state spaces. The frequency range produced is thus also limited per register. This implies that to extend their vocal range to lower or higher frequencies, animals need to be able to employ the adjacent register.

In human singing voice pedagogy, there is the typical case of amateur singers whose $f_{o}$ range is limited because they are ‘stuck’ in vocal register M1 [105,106, p. 116]. Their M1 voice is believed to be produced with excessive subglottal pressure and disproportionately low OQ values caused by increased VF adduction and TA muscle activation. This represents an extreme case of the cusp catastrophe scenario shown in figure 2B, where—at a given phonatory configuration—only one LC, supporting M1 is possible, which reduces the singer’s pitch range to the highest achievable $f_{o}$ in M1. Only a fundamental retraining of phonatory habits, enabling the production of M2 (and thus establishing at least some pathway to switch from M1 to M2), will increase the respective singer’s $f_{o}$ range and enable the production of higher pitched sounds.

While there is yet insufficient detailed knowledge about physiological control of vocal registers in non-human vertebrates, we hypothesize that the same underlying principle applies. The establishment of different and distinct physiological configurations for vocalization—enabling different vocal registers—allows animals to vastly extend their $f_{o}$ range.

## 5. Detecting vocal registers and transitions via vocal fold kinematics

Because we here define a vocal register based on the vibratory behaviour of the VFs, quantitative measures of VF kinematics provide the most direct evidence for registers and register transitions. However, registers also leave signatures in other physiological signals, including of course most notably the radiated sound, but also muscle activity and subglottal flow/pressure. In this section, we review the different techniques that have been used to detect vocal registers. Finally, we review in what taxa across the vertebrates vocal registers have been suggested based on different lines of observations.

### (a) Techniques to quantify vocal fold kinematics

Several techniques have been used to quantify the kinematics of VFs or analogous structures. Here, we discuss the two most commonly used techniques.

First, high-speed imaging can capture one-dimensional, two-dimensional or even three-dimensional shape changes of VFs over time within oscillatory cycles. High-speed imaging provides the highest data quality and confidence level to confirm vocal registers because it can accurately capture the time-varying VF shape during oscillation. When imaging at 1000s of frames per second, the sensors need large quantities of light and therefore, the technique remains very challenging to use *in vivo*, but is extensively used in isolated vocal organ studies *in vitro* and *ex vivo*. Another practical issue that makes this technique challenging *in vivo* is that two-dimensional high-speed imaging generates a large volume of data. These data are most often stored locally on the camera, so that only very brief segments can be imaged, which is a distinct disadvantage *in vivo*. Alternatively, all imaging data get flushed onto external storage, but this generates so much data that region of interest selection becomes time-consuming. A practical high-speed imaging system for VF imaging used in humans [107,108], non-human animals [109,110] and birds [111,112] is videokymography [113,114], which combines 25 Hz full-frame colour imaging with a line scan at 7200 Hz. This provides both positioning context and high-speed data, but only in one-dimension.

Second, a commonly used technique to quantify VF kinematics is electroglottography (EGG). In brief, EGG measures the tissue capacitance between two electrodes and can be used to estimate the relative contact area between vibratory structures (for detailed descriptions of the technique see [12]). Thus, no direct image of the VF shape is measured. However, because the waveform of the EGG signal during human speech and song production has been calibrated extensively against high-speed images, several landmark events, such as VF contacting and decontacting, can be recognized reliably. Therefore, the technique is used extensively in human voice research and clinical settings. However, also in comparative bioacoustics, the technique has been used in non-human primates [15,24] and [27] as well as in various bird species [111,112].

### (b) Evidence of vocal registers across the vertebrates

All voiced sounds produced by animals must occur in some register. Additionally, animals may have—like humans—access to multiple registers. In table 1, we listed examples of direct (VF kinematic) and indirect (acoustic and other) evidence of multiple registers in various taxa.

Based on high-speed imaging data, the vocal structures of humans, dogs [14,26], non-human primates [24], crows [21] and toothed whales [115] are capable (*in vitro*, *in vivo* or *in situ*) of producing multiple vocal registers. In all these species, registers greatly extend the fundamental frequency range outside the M1 register. In toothed whales, registers even added novel functionality to their vocalizations, as M0 vibration allows them to produce echolocation clicks for catching prey, while the other registers M1 and M2 are used for social communication [4]. Based on EGG *in vivo*, three additional occurrences of registers were suggested for non-human primates, i.e. Japanese macaques [24], Sykes’s monkeys [15] and a number of other New World monkeys (see [27]). When we expand the line of evidence to *in vitro* preparations of vocal organs, further testimony for different vocal registers has also been found using high-speed imaging and EGG in several mammals like house cats [25], dogs [16] and the pigeon *Columba livia* [23]. While this does not directly show that multiple vocal registers are used *in vivo* in these species, it shows conclusively that their vocal organ is capable of producing them. Finally, based on the acoustic signal only, vocal registers M0, M1 and M2 have been suggested to be used by dormice [19], African wild dogs [19], lambs [22] and Diana monkeys [20].

## 6. Using acoustics to detect vocal registers

Is it possible to detect the occurrence of multiple vocal registers based on acoustics alone? If so, this would be advantageous because the abovementioned techniques to measure VF kinematics are obviously much more complicated than recording sound. We would propose that of all vocal registers in this human-based classification, only the M0 register leaves a clear acoustic signature, because in M0 the period is long enough for each glottal excitation to effectively die out before the next acoustic excitation occurs [116]. Therefore, pulsatile acoustic signals are good candidates for M0 vibration that have been confirmed in several cases (see above).

### (a) Vocal register footprints in the acoustic signal

First, when two vocalizations of the same individual have very distinct $f_{o}$ or spectral ranges: Established examples are different vocalizations in Odontocetes [4] or the coo call in Japanese macaques [24]. However, note that it is also possible that these distinct register candidates are instead produced by different vibrating structures, e.g. the grunt of koalas (velar folds) [117], the phee call in marmosets (vocal membranes) [118] or the agonistic calls of bats (vestibular folds) [119].

Second, an additional salient feature can be transitions between two registers. In humans, an abrupt frequency jump or abrupt change in spectral composition can be an identifying characteristic of a register transition [11,39,41], a notion that may generalize to voiced sound production at large. Abrupt transitions of fundamental frequency and/or spectral composition are found in many animal vocalizations, including non-human primates [18,120], canines [121–123], cats [124], deer [125], elephants [126], rodents [127–136], bats [137], baleen whales [138–140], dolphins [141], manatees [142], as well as frogs [143,144], lizards [145] and birds [80,146–149]. Given the common occurrence of these abrupt changes across vertebrates, is it thus likely that vocal registers are much more common than previously thought. However, these above jumps require case-by-case consideration to conclusively be considered a vocal register.

### (b) Established false detections of vocal registers

The above theory suggests that registers can leave clear traces in acoustics. However, there are at least two established cases of incorrect detection of a register transition based on acoustics , which complicates matters. One is a false positive and one a false negative.

One cause for abrupt register transitions is smooth and often small changes in VF positioning that are controlled by muscles. There is only little quantitative experimental data—even in humans—on muscle activation during voice production. Small changes in force production by TA, CT and PTA muscles in the larynx of humans [55,150] and dogs [14,26], or expiratory pressure [16] via diaphragm and intercostal muscles, are known to cause abrupt transitions between vocal registers. However, how fast is abrupt? In vocal production systems, the laryngeal and syringeal muscles of mammals and birds can achieve such speeds that they can control parameters on millisecond scales [151–153]. For example, when a bird produces an $f_{o}$ of 500 Hz, the period of one oscillation is 2 ms. The superfast muscles controlling the syrinx can achieve full force in only 5–10 ms in finches [154] and 1–2 ms in starlings [151], and can thus change parameters within a few or even a single oscillation cycle(s). This is hard to distinguish in the oscillogram of the sound, and certainly in the coarse time resolution of the spectrogram. Thus, such fast changes can give the impression of a bifurcation while in fact the animal is controlling $f_{o}$ using fast muscle contractions [155]. Because the proteins underlying superfast muscle performance are evolutionarily ancient [153], the fibre type is probably commonly present in the larynx and syrinx across vertebrates. Because superfast fibres trade off force for speed, they are mostly found in smaller animals [156]. Thus, superfast modulation of control parameters in smaller animals can provide a false positive: there may seem to be a register transition or other bifurcation when there is none.

The other case comes from human singing. When vocalization encompasses multiple vocal registers, the transitions between them can either be abrupt (as in ‘voice cracks’ of pubescent boys or in alpine yodelling, for example), or smooth. Particularly in human classical/operatic singing, a major goal is to *avoid* abrupt and salient alterations of $f_{o}$ and spectral characteristics, forcing the singers to ‘mix’ or ‘blend’ the vocal registers in a seamless fashion (recall figure 2). To what extent this is achieved through adaptations of vocal tract resonance characteristics [83,157] and/or modifications of the laryngeal vibratory states [92,158] is still subject to debate. Either way, this must be achieved by different activation of muscles that achieve a different path through the motor control space, but how this is done remains largely unknown. Thus, register blending—which might also occur in non-human vocalizations—can provide a false negative: there seems to be no register transition or other bifurcation while there is one.

## 7. Conclusions

We suggest that vocal registers are a fundamental trait of vertebrate voice production physiology. Vocal registers are enabled by nonlinear dynamics of the sound production apparatus, powered by the entrainment possibilities of the sound-generating tissue eigenmodes and coupled supraglottal and subglottal acoustic resonators. We suggest that $f_{o}$ range separation between a species’ different call types and discontinuities between two nearly periodic vibratory states—i.e. frequency jumps and/or abrupt alterations of the radiated acoustic spectrum—can be acoustic hallmarks of register transitions. Occurrence of such salient phenomena in a species’ vocalization acoustics warrants deeper empirical investigation of VF kinematics. The possibility for producing vocal registers seems intrinsic to the MEAD mechanism found in several vocal organs across the vertebrates. Vocal registers expand a species’ signal diversity in vocal communication by discretizing the acoustic output into distinct call types and extending the $f_{o}$ range used for vocalization.

## Data Availability

Supplementary material is available online at [159].
